# Characteristics and clinical associations of late gadolinium enhanced cardiovascular magnetic tesonance in lamin A/C, cardiac troponin T and myosin binding protein C gene mutation related cardiomyopathies

**DOI:** 10.1186/1532-429X-15-S1-P150

**Published:** 2013-01-30

**Authors:** Jennifer Franke, Sebastian A Seitz, Hassan Abdel-Aty, Mohamed A Abdelrazek, Dirk Lossnitzer, Hugo A Katus, Florian Andre

**Affiliations:** 1 Department of Cardiology, University of Heidelberg, Heidelberg, Germany; 2 Radiology, Cairo university, Faculty of Medicine, Cairo, Egypt

## Background

Dilated cardiomyopathy (DCM) is a common cause of heart failure (HF) and the most common diagnosis in patients referred for cardiac transplantation. Genetic studies in DCM have identified mutations in over 30 genes. To date, there is only scarce data regarding the interdependencies between the various genotypes and their impact on cardiac morphology and myocardial tissue appearance. Since non-contrast cardiac magnetic resonance (CMR) is considered the reference method for morpho-functional analysis and late gadolinium enhanced (LGE) CMR method of choice for myocardial tissue assessment, we sought to investigate the characteristics of LGE in asymptomatic and symptomatic carriers of known cardiomyopathy mutations and their clinical associations.

## Methods

Thirtyeight carriers of lamin A/C (LMNA), cardiac troponin T (TNNT2) or myosin binding protein C (MYBPC3) gene mutations (LMNA in 5, TNNT2 in 3, MYBPC3 in 27, MYBPC3+TNNT2 in 3 patients) were investigated with a 1.5T clinical scanner (Philips Achieva). Short axis slices covering entirely both ventricles were acquired using standard SSFP-sequences for measurement of LV and RV volumes and ejection fraction (EF). Focal myocardial fibrosis was assessed in LGE CMR images acquired 10 minutes after i.v. injection of 0.2 mmol/kg Gd-DTPA (Magnevist). Standard SSFP and LGE CMR images were assessed by different observers who were blinded to measurement results and clinical data. Associations between clinical characteristics, gene mutation and LGE were analyzed.

## Results

LGE was present in 19 of 30 (63%) MYBPC3 carriers, all 6 (100%) TNNT2 carriers and 1 of 5 (20%) LMNA carriers. Midwall patterns of LGE located in the basal and/or mid-ventricular septal wall were the most commonly seen patterns in all gene mutations (57% of all LGE positive patients). The presence of LGE was positively associated with age (rs=0.379; p=0.019). No further significant correlations were found between the presence, pattern or location of LGE and gender, cardiovascular risk factors, baseline systolic LV function or conduction abnormalities. Of all patients with known mutations, 24/38 (63%) were asymptomatic. No significant difference in LGE features was found between symptomatic and asymptomatic mutation carriers (10/14 vs. 12/24; p=ns).

## Conclusions

LGE, predominantly presenting with basal and/or mid-ventricular septal midwall patterns, is a common finding in cardiomyopathy patients with known gene mutations. Its presence seems to be independent of the symptomatic stage of the disease or its phenotypic manifestation.

## Funding

none

